# Nematicidal Properties of Chitosan Nanoformulation

**DOI:** 10.2478/jofnem-2023-0033

**Published:** 2023-08-24

**Authors:** R. Mouniga, B. Anita, A. Lakshmanan, A. Shanthi, G. Karthikeyan

**Affiliations:** Tamil Nadu Agricultural University, Coimbatore (Tamil Nadu), India.

**Keywords:** Chitosan nanospheres, *Meloidogyne incognita*, particle size, tomato, biochemistry

## Abstract

Chitosan is the second most abundant bio-polymer available in the world, second only to cellulose. It is found in crustaceous shells, e.g., those of crabs, shrimps, prawns, and fungi, as well as insect exoskeletons. The use of nanoformulations for the management of pests and diseases is receiving increased interest with the advancement of nanotechnology. Here, chitosan nanospheres were obtained from chitosan using the ionic gelation technique. The nanoformulations obtained were characterized using a particle size analyzer, Fourier transform infrared spectroscopy, and a transmission electron microscope. The efficacy of chitosan nanospheres in suppressing the root-knot nematode *Meloidogyne incognita* was studied. The particle size of nanospheres formulated for this study was 380.2 nm, with a polydispersity index (PI) of 0.4 and Zeta potential of 45.7 or 50.9 mV at pH 5.2. The chitosan nanospheres were spherical and the particles did not agglomerate. FTIR spectra of the chitosan nanospheres peaked at 3334 cm^−1^, thereby indicating the stretching of the OH and NH group. In In-vitro studies, chitosan nanospheres showed significant nematicidal activity against *M. incognita*. Under pot culture conditions, chitosan nanospheres (1%- active compound chitosan) at 2ml/plant decreased the nematode population in roots or soil. Compared to the control, the number of galls was reduced by 83.68%, the number of egg masses by 83.85%, the number of adult females by 66.56%, and the number of second-stage juveniles by 73.20%. In a field experiment, application of chitosan nanospheres (1%) was followed by a 18.75% increase in fruit yield compared to the non-treated control.

Biopolymers are naturally occurring materials formed during the life cycle of plants, animals, bacteria, and fungi (Yadav et al., 2015). Biopolymers are easily biodegradable because they include oxygen and nitrogen atoms. Through biological processes, the biopolymers are naturally recycled. Worldwide, chitosan is the second most abundant biopolymer, second only to cellulose. Through enzymatic and chemical deacetylation processes, chitin is converted to chitosan (Kafetzopoulos et al., 1993). Chitosan was first discovered in mushrooms by Henri Braconnot in 1811 (Periayah et al., 2016). It is obtained from crustaceous shells, e.g., from the exoskeletons of crabs, shrimps, prawns, fungi, and insects. Chitosan consists of N-actetyl D-glucosamine and β-(1-4) D-glucosamine. Glucosamine (GlcN) is product of the decomposition of chitosan by the chitosanase enzyme (Jung and Park, 2014). Chitosan is considered a cationic polymer, and due to its biocompatibility, nontoxicity, and bio-degradability properties, it is used in agricultural, medical, biotechnological, environmental, and industrial applications (Naveed et al., 2019). It is also known to possess antifungal, antibacterial, antiviral and antinematicidal properties (Goy et al., 2016).

Tomato (*Solanum lycoperiscum*) is the second most-abundant vegetable crop next to potato, with an annual production of 182.3 million tons on 4.85 million hectares. The southern root-knot nematodes, *Meloidogyne incognita*, *M. javanica* and *M. arenaria*, are the most frequent nematode species infecting tomato. Nematode infection can predispose the plant to fungal infection, leading to disease complexes causing considerable yield losses (Khan and Sharma, 2020). Ganaie and Khan (2011) studied the interactive effect of the root-knot nematode *M. incognita* and the root pathogenic fungi *Fusarium solani* on tomato. They concluded that inoculation of root-knot nematode *M. incognita,* followed by inoculation of *F. solani*, caused more severe damage on tomato than either pathogen alone. When chitosan is applied to soil, it is converted into chitin (a polysaccharide), and then into chitobiose (a disaccharide). Chitobiose damages the eggs and cuticles of young nematodes, which have chitin in their composition (Gortari et al., 2008; Abd El-Aziz and Khalil, 2020). The release of toxic chemical compounds during decomposition has a lethal effect on the second-stage juveniles of *M. incognita* and on nematode multiplication (Asif et al., 2017). During chitin hydrolysis, ammonia concentrations increase, causing death in nematodes (Spiegel et al., 1987). Chitosan induces signaling molecules in plants, such as the specific cellular receptor that is transduced by secondary messengers. These secondary messengers include Reactive Oxygen Species (ROS), H_2_O_2_, Ca^2+^, nitric oxide, and phytohormones (Hidangmayum et al., 2019). In several studies, chitosan has been used as a nanocarrier for encapsulated drugs or active compounds, delivering them into a specific place and providing controlled release (Yanat and Schroen, 2021).

Nanospheres are nanostructures formed by a dense polymeric matrix. The drugs are dispersed in the matrix-type structure of the nanospheres. Nanospheres are prepared using several polymers like cellulose, chitosan, and Poly (d, l-lactic acid). The size range of nanospheres is between 10 and 200 nm in diameter. Nanospheres are amorphous or crystalline in nature (Singh and Sharma, 2010). Embedding the desired compounds in nanospheres protects the drugs from enzymatic and chemical degradation. Chitosan nanoparticles are used as nanopesticides and carriers of fungicides, insecticides, herbicides, plant hormones, elicitors, and nucleic acids (Zhang et al., 2003; Dei Lam et al., 2006). Because of their small size and high contact area, chitosan nanoparticles can easily penetrate and permeate into the membrane of phytopathogens or plant tissues, resulting in increased control or defense response activity.

Alfy et al. (2020) reported that a chitosan nanoparticle at 2000 ppm was the most efficient biopolymer in controlling the root-knot nematode, *M. incognita*, in tomato. Nanochitosan at 2000 ppm decreased the egg hatching rate by up to 95.3% and caused 97.2% juvenile mortality after 72 hr of exposure. It increased plant growth parameters and, compared to the control, decreased the nematode population in soil by 78% and in the roots by 98%. Nematode management remains challenging due to the limited availability of nematicides.

Human health concerns and environmental considerations call for environmentally-friendly alternatives that can be integrated into existing practices. With this background in mind, a study was undertaken to assess the efficacy of chitosan nanospheres against root knot nematode, *M. incognita.* Our objectives were to formulate and characterize of chitosan nanospheres and determine their efficacy on *M. incognita* under in-vitro, field and pot culture conditions.

## Materials and Methods

### Preparation of chitosan nanospheres

Commercial-grade high-molecular-weight chitosan was used in the study (obtained from Panvo pvt ltd. Chennai). Chitosan nanospheres were prepared using the ionic gelation method (Youssef and Masry, 2018). 1g of crude chitosan was dissolved in 1% glacial acetic acid and stirred for 24 hr in a magnetic stirrer at 500 rpm (Spinit TM Digital Magnetic stirrer). After 24 hr of stirring, one part 0.7 mg/ml Sodium Tripolyphosphate (TPP) was added dropwise to 3 parts of 1% chitosan acidic solution and again allowed for 2 hr, stirring at 500 rpm. The pH of the prepared solution was checked using a pH meter and later sonicated in an Ultraprobe sonicator (Ultra Sonics instruments) with an amplitude of 35%, pulse 10 seconds, and temperature of 35ºC, for seven minutes.

In a second experiment, 5 ml of surfactant 2% Tween 80 was slowly added to 3 parts of 1% chitosan acidic solution at 45ºC. The mix was stirred for 2 hr in a magnetic stirrer at 500 rpm. One part of TPP was added to three parts of 1% chitosan-acetic acid solution. The sample was then homogenized in a pressurized homogenizer (Homeostat instrument) for 5 minutes and ultrasonicated for 15 min.

### Characterization of chitosan *nanospheres*

The particle size and polydispersity index (PI) of the synthesized chitosan nanoparticles were measured in a particle size analyzer (HORIBA SZ-100, Japan). Their stability was determined using zeta potential. The size and shape of the chitosan nanospheres were characterized using transmission electron microscopy (TEM). The functional groups of biomolecules present in the chitosan nanospheres were identified using Fourier infrared spectroscopy (FTIR).

### Bioefficacy of chitosan nanospheres against root knot nematode, *M. incognita*

Pure cultures of root-knot nematode, *M. incognita*, were obtained at Department of Nematology, TNAU, Coimbatore. Concentrations of 100 ppm, 500 ppm, 1000 ppm and 5000 ppm were prepared from the 1% chitosan nanosphere suspensions in sterile (100 ml) water. Each concentration was sonicated in a water bath sonicator (POWERSONIC 510) for 20 minutes before testing them against *M. incognita*.

### Egg hatching test

Egg masses of *M. incognita* were sterilized in 0.5% sodium hypochlorite solution for 1 minute. The culture was maintained at Department of Nematology, Glass house, TNAU, CBE. Egg masses were collected from nematode-infested root. The experiment was repeated 3 times. 2 ml of each concentration suspension was placed in a 5-cm diameter petri dish. One egg mass of M. incognita was placed into the liquid in the petri dish. The experiment was conducted in a randomized complete block design, with five treatments and four replications. Numbers of hatched juveniles were observed at different time intervals *viz.,* 24 hr, 48 hr and 72 hr.

### Juvenile mortality test

Freshly hatched second-stage juveniles (J2) were used for juvenile mortality studies. For each different concentration of the chitosan nanospheres, 1ml of the suspension was placed in a 5-cm diameter petri dish, to which 100 infective juveniles (J2) were added (the 1ml suspension containing nematodes were counted with the help of a counting dish under a stereo zoom microscope). The five treatments were replicated four times and arranged in a randomized complete block design. All 100 juveniles were examined, and the number of dead juveniles were recorded after 24 hr, 48 hr and 72 hr.

### Efficacy of chitosan nanospheres on Root knot nematode in pots in a greenhouse (in-vitro conditions)

A greenhouse experiment in pots (3 kg of soil; pot size 20.7 × 11 × 16.4 cm) was conducted to study the effect of different concentrations of chitosan nanospheres against *M. incognita*. Seedlings purchased from the vegetable nursery at Thondamuthur (15 days old; Shivam hybrid) Coimbatore-03 were used for pot culture experiments. The 15-day-old seedlings were transplanted into a sterilized pot mixture, where they were inoculated with infective juvenile nematodes at a rate of 2/gm of soil. One week after inoculation, chitosan nanospheres were applied as a soil drench. On the 45th day after nematode inoculation, plants were uprooted, and plant shoot lengths and root weights were determined. The nematode populations were counted based on the gall numbers (Heald et al., 1989) and egg masses on the roots. In total, 10 treatments and three replications were arranged in a randomized complete block design.

The treatments included:

1. Chitosan nanospheres (1%) 1ml/plant.
2. Chitosan nanospheres (1%) 2ml/plant.
3. Chitosan nanospheres (1%) 3ml/plant.
4. Chitosan (1%) 1ml/plant.
5. Chitosan (1%) 2ml/plant.
6. Chitosan (1%) 3ml/plant.
7. Velum prime 1ml/plant-34.6% Fluopyran
8. Purpureocillium lilacinum 1g/plant.
9. Chitosan nanospheres (1%) 3ml without nematodes.
10. Untreated control.

### Effect of chitosan nanospheres against root knot nematode, *M. incognita* under field conditions

A *M. incognita*-infested field was selected near Karadimadai Village, Coimbatore, India. Seven treatments were applied as soil drenches to one week-old tomato seedlings. On the 45th day after planting a second set of treatments were applied. Treatments were applied to three replicate plots with 40 plants each, arranged in a randomized block design. Plant growth parameters and nematode population density in soil and roots were observed 90 days after application. Fruit yields were measured for each treatment.

The following treatments were used:

T1- Chitosan nanospheres 1% (5ml/plant).
T2- Chitosan nanospheres 2% (5ml/plant).
T3- Chitosan 1% (5ml/plant).
T4- Chitosan 2% (5ml/plant).
T5- Velum prime (34.46% fluopyran)- 500 ml/acre.- 0.0005 metric
T6- Carbofuran 3G 1kg a.i/ha.
T7- Untreated control.

### Statistical analysis

The data obtained from the above-mentioned experiments were subjected to statistical analysis following the method formulated by Panse and Sukhatme (1967). The obtained data were run in Aggress software with single-factor analysis to obtain the results.

## Results

### Formulation and characterization of chitosan nanospheres

**Figure 1:**
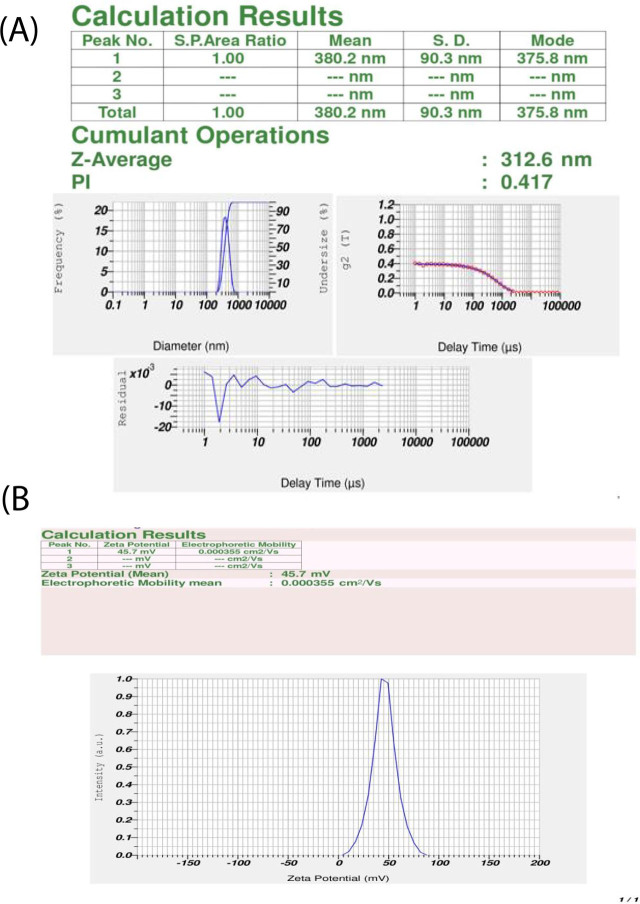
(A) Particle size of chitosan nanospheres. (B) Zeta potential of chitosan nanospheres.

1g chitosan was dissolved in 1% acetic acid in the presence of Tween 80 and TPP. The prepared solution was homogenized in a pressurized homogenizer for 5 minutes. The homogenized solution yielded a size of 380.2 nm with a PI of 0.4 (Fig. 1). The present study proved that synthesized chitosan nanospheres using sodium tripolyphosphate and Tween 80 were highly stable, as measured by their zeta potential value of +49.7 mV (Fig. 1b). The zeta potential of the chitosan nano formulation obtained in this study was well above +30 mV, indicating high stability.

### Transmission Electron Microscope (TEM)

**Figure 2:**
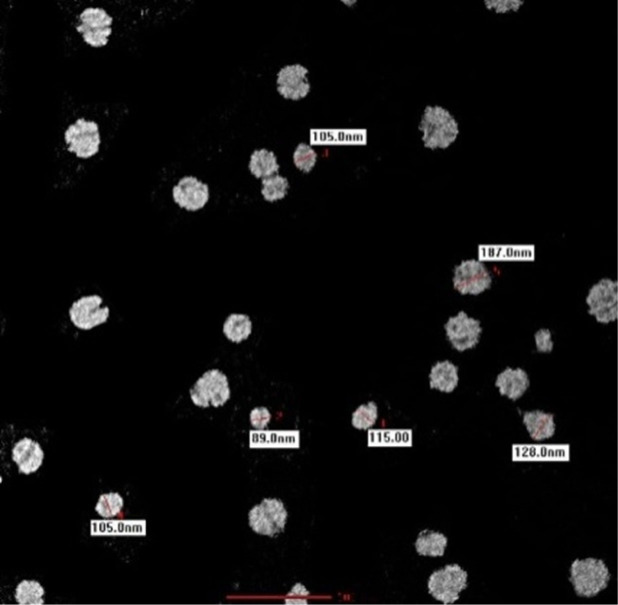
Transmission Electron Microscope (TEM) micrograph of chitosan nanospheres.

TEM micrography was used to study the morphology, shape, and size of the synthesized chitosan nanospheres. The nanospheres were predominately spherical, with no agglomerates. The average size of each nanosphere was 89.0 nm (Fig. 2).

### FTIR (Fourier Transform Infra-Red Spectroscopy)

**Figure 3:**
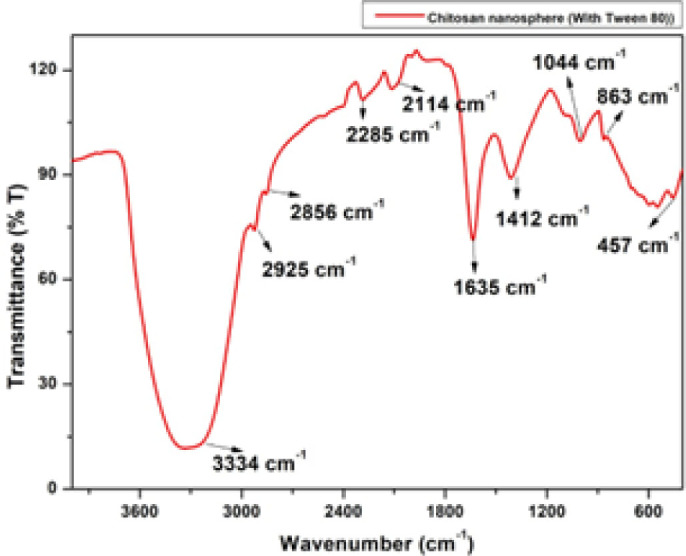
FT-IR spectroscopy of 1% chitosan nanospheres formulation.

The chitosan nanospheres obtained with the above method were analyzed using Fourier Transform Infra–red Spectroscopy (FTIR), which was used to study the chemical interaction between chitosan and sodium tripolyphosphate molecules. The peak at 3334 cm^−1^ showed a stretching of the OH and NH group. Peaks at 2925 cm^−1^ and 2856 cm^−1^ show the stretching of the CH group. A peak of 2285 cm^−1^ represented N=C=O stretching. A peak at 2114 cm^−1^ showed N=C=S stretching and a peak at 1635 cm^−1^ explained the stretching of C=N. The peak at 1412 cm^−1^ showed bending of the O-H group and the peak at 1004 cm^−1^ revealed the stretching of C-F group (Fig. 3).

### Bio-efficacy of chitosan nanospheres against Root knot nematode *M. incognita* under in-vitro conditions

**Figure 4:**
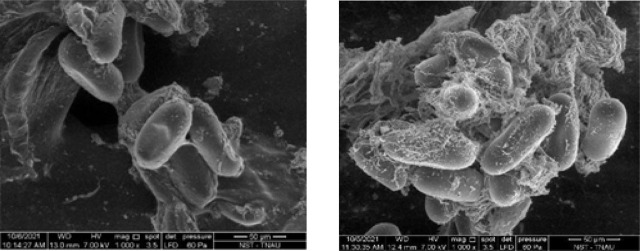
Scanning Electron Microscope images of chitosan nanospheres treated eggs (left side) and chitosan nanospheres untreated eggs (right side).

To assess the efficacy of chitosan nanospheres on the hatching of *M. incognita* eggs, different concentrations of chitosan nanospheres were used. Among the concentrations tested, chitosan nanospheres at 1000 ppm and 5000 ppm completely inhibited egg hatching compared to the control. Exposure of *M. incognita* egg masses to chitosan nanospheres at 500 ppm concentration resulted in a 68.75% decrease in egg hatching compared to the untreated control 72 hr after treatment (Table 1). At this concentration, hatching of eggs was observed, but all the hatched juveniles from the eggs were found dead. A scanning electron microgram (x1000 magnification) of treated egg masses revealed degradation of the gelatinous matrix (Fig. 4). The first-stage juveniles found within the treated eggs were deformed.

**Table 1.** Effect of chitosan nanospheres on the egg hatching of *M. incognita*

**Treatments**	**Number of hatched juveniles (Mean of four replications)**		

	**24 h**	**48 h**	**72 h**
	**Mean and transformed value**	**Mean and transformed value**	**Mean and transformed value**
Chitosan nanospheres at 100 ppm	12.50^b^ (3.31)	16.50^b^ (4.84)	41.50^b^ (6.29)
Chitosan nanospheres at 500 ppm	10.50^b^ (3.09)	14.16^b^ (3.61)	26.50^b^ (4.79)
Chitosan nanospheres at 1000 ppm	0.00^a^ (0.70)	0.00^a^ (0.70)	0.00^a^ (0.70)
Chitosan nanospheres at 5000 ppm	0.00^a^ (0.70)	0.00^a^ (0.70)	0.00^a^ (0.70)
Control- tap water	38.83^c^ (6.21)	61.66^c^ (7.85)	84.80^c^ (17.27)
SEd	0.72	0.46	1.12
CD (p=0.01%)	2.29	1.48	3.88

* Figures in parentheses are square root transformed value. In a column, means followed by common different from each other at 1% level by DMRT.

Different concentrations of 1% chitosan nanospheres *viz*., 100 ppm, 500 ppm, 1000 ppm and 5000 ppm were tested against infective juveniles (J2). Among the above-mentioned concentrations, chitosan nanospheres at 5000 ppm caused 100% juvenile mortality within 24 hr (Table 2). At the 1000-ppm concentration, the infective juvenile mortality was lower, with a maximum of 46.25% mortality observed after 72 hr of exposure.

**Table 2.** Effect of chitosan nanospheres on *M. incognita* juvenile mortality

**Treatments**	**Juvenile mortality in percentage (Mean of four replications)**		

	**24 h**	**48 h**	**72 h**
Chitosan nanospheres at 100 ppm	13.00^b^ (5.40)	21.5^c^ (3.47)	26.5^c^ (5.10)
Chitosan nanospheres at 500 ppm	27.25 ^b^ (5.14)	31.25^b^ (5.67)	28.25^c^ (5.26)
Chitosan nanospheres at 1000 ppm	29.00 ^b^ (5.85)	36.00^b^ (5.96)	46.25^b^ (6.70)
Chitosan nanospheres at 5000 ppm	100.00^a^ (10.02)	10.00^a^ (10.02)	100.00^a^ (10.02)
Control	0.00^c^ (0.707)	0.00^d^ (0.70)	0.00^d^ (0.70)
SEd	1.52	0.72	0.55
CD (p=0.01%)	4.48	2.14	1.64

* Figures in parentheses are square root transformed value. In a column, means followed by common alphabet are significantly different from each other at 1% level by DMRT.

### The effect of chitosan nanospheres on root knot nematode *M. incognita* in tomato under pot culture and field conditions

**Figure 5:**
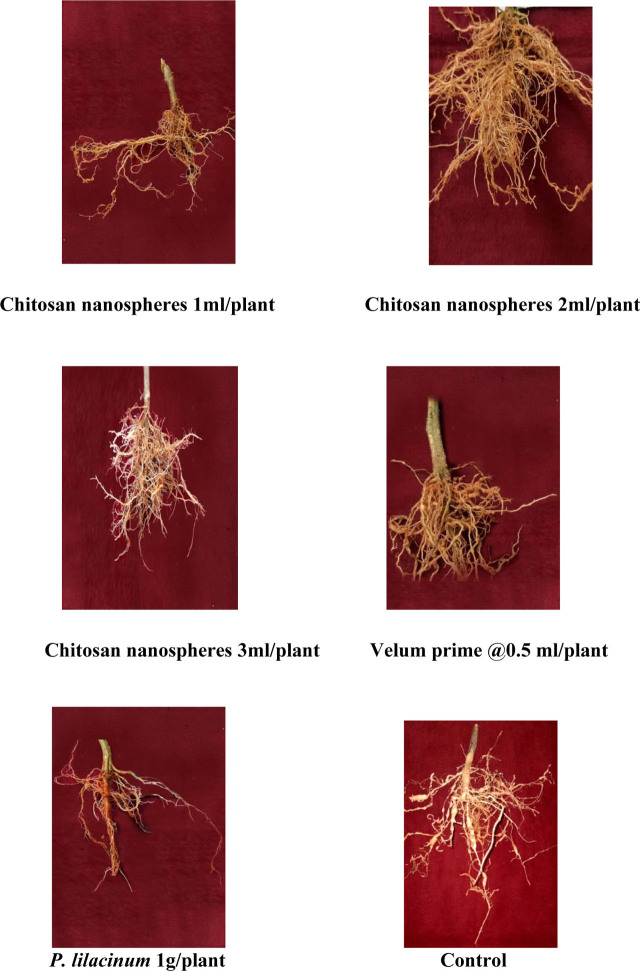
Efficacy of different doses of chitosan nanospheres (1%) on *M. incognita* under pot culture conditions.

In-vitro results revealed that chitosan nanospheres reduced nematode population in roots and soil. Application of chitosan nanospheres at 2ml/plant decreased root galls by 83.68%. Application of chitosan nano formulation at 2ml/plant decreased the number of egg masses by 83.85%. The chitosan nanosphere formulation at 2ml/plant registered the lowest number of adult females, with the highest percent reduction of 66.56 % (Table 3). The highest reduction of infective juveniles (73.20%) was recorded with the 2ml/plant formulation (Fig. 5). In field experiments, the chitosan nanosphere formulation (2%) at 5ml/plant decreased galls by 92.47%. Furthermore, the fruit yield was higher by 18.75% in plots treated with chitosan nanospheres (Table 4).

**Table 3.** Effect chitosan nanospheres against root knot nematode, *M. incognita* under pot culture conditions

**Treatments**	**Mean of three replications**			

	**Number of galls/5g of roots**	**Number of egg masses/5g of roots**	**Number of females/5g of roots**	**Number of J2/100 cc of soil**

	**Mean and transformed value**	**Mean and transformed value**	**Mean and transformed value**	**Mean and transformed value**
Chitosan nanospheres 1ml/plant+ *M. incognita*	13.30^a^ (3.51)	11.67^bcd^ (3.32)	17.67^cd^ (4.25)	48.60^bc^ (12.06)
Chitosan nanospheres 2ml/plant+ *M. incognita*	10.00^a^ (3.12)	6.00^b^ (2.53)	14.60^b^ (3.87)	28.30^ab^ (5.36)
Chitosan nanospheres 3ml/plant+ *M. incognita*	15.00^a^ (3.87)	7.33^bc^ (2.73)	17.60^cd^ (4.25)	40.30^bc^ (6.38)
Chitosan 1ml/plant+ *M. incognita*	18.00^a^ (4.28)	19.00^c^ (4.41)	22.30^f^ (4.76)	62.00^bcd^ (7.90)
Chitosan 2ml/plant + *M. incognita*	17.00^a^ (4.25)	10.00^bcd^ (3.22)	16.67^c^ (4.13)	46.70^bc^ (6.86)
Chitosan 3ml/plant + *M. incognita*	17.60^a^ (3.34)	10.66^bcd^ (3.30)	17.90^d^ (4.19)	44.70^bc^ (6.66)
Velum prime 0.5ml/plant + *M. incognita*	25.67^a^ (4.87)	15.67^de^ (3.95)	19.30^e^ (4.44)	32.67^bc^ (5.75)
*P. lilacinum* 1g/plant+*M. incognita*	17.67^a^ (4.25)	13.39^cde^ (1.37)	18.67^e^ (4.44)	50.60^bc^ (7.14)
Chitosan nanospheres without nematode Inoculums	0.00^a^ (0.70)	0.00^a^ (0.70)	0.00^a^ (0.70)	0.00^a^ (0.70)
Untreated control	61.30^c^ (7.83)	37.16^f^ (6.04)	43.67^g^ (6.63)	105.60^cd^ (10.21)
SEd	0.96	0.47	0.06	2.26
CD (p=0.01%)	2.73	1.36	0.18	6.44

* Figures in parentheses are square root transformed value. In a column, means followed by common alphabet are significantly different from each other at 1% level by DMRT.

**Table 4.** Effect of chitosan nanospheres against root knot nematode, *M. incognita* under field conditions

**Treatments**	**Mean of three replications**					

	**Number of galls/5g of roots^1^**	**Number of egg masses/5g of roots^1^**	**Number of females/5g of roots^1^**	**Number of J2/100 cc of soil^1^**	**Fruit weight (kg)/plot^2^**	**Fruit weight (tonne)/ha^2^**

	**Mean and transformed value**	**Mean and transformed value**	**Mean and transformed value**	**Mean and transformed value**	**Mean and transformed value**	
Chitosan nanospheres (1%)-5ml/plant	5.61^ab^ (2.29)	3.34^b^ (1.82)	18.31^b^ (4.26)	50.63^c^ (7.11)	95^a^ (18.75)	14.25
Chitosan nanospheres (2%)-5ml/plant	3.31^a^ (1.81)	0.99^ab^ (1.14)	17.38^b^ (4.16)	35.00^b^ (5.90)	89.5^b^ (11.87)	13.42
Chitosan (1%) - 5ml/plant	11.25^b^ (3.10)	3.62^b^ (1.93)	20.90^b^ (4.54)	58.54^e^ (7.61)	86^bc^ (7.5)	12.90
Chitosan (2%)-5ml/plant	4.91^ab^ (2.30)	1.88^ab^ (1.52)	17.79^b^ (4.19)	48.75^b^ (6.92)	83^cd^ (3.75)	12.45
Velum prime 500ml/acre	2.83^a^ (1.60)	0.09^a^ (0.75)	11.42^a^ (3.35)	30.45^a^ (5.47)	99^a^ (23.75)	14.85
Carbofuran 3G- 1kg ai/ha	7.32^b^ (2.95)	1.43^ab^ (1.35)	23.20^b^ (4.78)	54.23^d^ (7.34)	89^b^ (11.25)	13.35
Untreated control	44.00^c^ (0.62)	22.3^c^ (4.77)	57.25^c^ (7.57)	165.80^f^ (12.73)	80^d^	12.00
SEd	0.46	0.37	0.37	0.10	2.28	
CD (p=0.05%)	1.00	0.80	0.80	0.22	4.90	

* ^1^ and ^2^-Figures in parentheses are square root transformed value and increased over control respectively. In a column, means followed by common alphabet are significantly different from each other at 1% level by DMRT.

## Discussion

Synthesis and characterization of chitosan nanoformulation and its potential antinemetic and antifungal effects are discussed. Per the observations of Budi et al. (2020), the average particle size of chitosan nanospheres was within the limit of 70 nm, with a Polydispersity Index of 0.3. In the present study, the particle size of chitosan nanospheres after using Tween 80 and sodium tripolyphosphate was found to be the 380.2 nm, with a PI of 0.4. Karava et al. (2020) determined that the optimum size of chitosan nanospheres was 150 nm, with a PI value below 0.6. A PI value between 0 and 0.5 indicates homogeneous particles and the PI value beyond 0.5 indicates particles in a heterogeneous condition (Danaei et al., 2018). The PI value of the nanoformulation obtained in our study was found to be within the range of 0.5, indicating nanoparticles of a homogenous nature.

High stability in synthesized chitosan nanospheres is considered to be a desirable characteristic. The present study proved that the chitosan nanospheres that were synthesized using sodium tripolyphosphate and Tween 80 were highly stable, as measured by their zeta potential value of +49.7 mV. The chemical interaction between chitosan and sodium tripolyphosphate molecules was studied using Fourier-transform infrared spectroscopy. The peak at 3334 cm^−1^ showed a stretching of the OH and NH groups. The findings of present study were similar to the observations of Youssef and Masry (2018), where observations made through transmission electron microscopy and field emission scanning electron microscopy revealed a spherical shape in the chitosan nanospheres with sizes of 89.0 nm - 187.0 nm and no agglomeration of particles. Youssef and Masry (2018) and Mohammadpour et al. (2011) also observed similar spherical shape of chitosan nanospheres.

Different concentrations *viz.,* 100 ppm, 500 ppm, 1000 ppm and 5000 ppm prepared from the 1% chitosan nanospheres solution were evaluated for their respective nematicidal properties. Exposure of egg masses to chitosan nanospheres at 1000 and 5000 ppm completely inhibited egg hatching and increased the death rate of juveniles. Alfy et al. (2020) reported that chitosan nanospheres concentrated at 2000 ppm inhibited *M. incognita* egg hatching by 95.3% after 72 hr of exposure and caused a 77.5% mortality rate in juveniles. Similar to the treated eggs, the infective juveniles exposed to chitosan nanospheres were found dead with deformed internal body parts. This was probably due to the interaction of positively charged chitosan with the negatively charged nematode, leading to the leakage of proteinaceous constituents as reported by Rabea and Badawy (2003).

Adding chitosan to the soil increases the population of chitinolytic microogranisms, which produce enzymes that convert chitin (a polysaccharide) to chitobiose (a disaccharide). This destroys the eggs and cuticles of young juveniles, which contain chitin (Abd El-Aziz and Khalil, 2020). Because of chitosan's eliciting activity and ability to generate systemic resistance in the plant, as well as the release of different toxic chemical compounds during decomposition, it is lethal to *M. incognita* second stage juveniles and inhibits nematode multiplication (Asif et al., 2017).

In the present study, the methodology of formulating chitosan nanospheres was standardized. The chitosan nanosphere formulations had both direct and indirect effect on root-knot nematodes. They degraded the chitin layer of nematode eggs and juveniles and caused death. Indirectly, they induced systemic resistance in plants, thereby reducing nematode infection. As chitosan nanospheres are synthesized from a biological source, the formulation is environmentally friendly and does not leave any toxic residues in the ecosystem.

## Abbreviations

TPP: Sodium Tripolyphosphate
PSA: Particle Size Analyzer
TEM: Transmission Electron Microscope
FTIR: Fourier Transform Infra-Red Spectroscopy
