# Erratum to: Nematicidal Properties of Chitosan Nanoformulation

**DOI:** 10.2478/jofnem-2025-0022

**Published:** 2025-06-11

**Authors:** R. Mouniga, B. Anita, A. Lakshmanan, A. Shanthi, G. Karthikeyan

In the version of this article, originally published 2023 on August 24, 2023, the authors state in the introduction on page 2: ‘When chitosan is applied to soil, it is converted into chitin (a polysaccharide), and then into chitobiose (a disaccharide).’ This statement implies that chitosan converts back to chitin when in the soil, which is wrong. The correct statement from the cited source article states ‘Also, addition of chitin or chitosan to the soil enhanced the population of chitinolytic microorganisms (relating to the enzyme converting chitin (a polysaccharide) into chitobiose (a disaccharide) that ruin the eggs and cuticles of young nematodes which have chitin in their composition.

